# Joint Modeling of Longitudinal Outcome and Competing Risks: Application to HIV/AIDS Data

**DOI:** 10.34172/jrhs.2023.106

**Published:** 2023-03-30

**Authors:** Khadijeh Najafi Ghobadi, Hossein Mahjub, Jalal Poorolajal, Ebrahim Shakiba, Kaivan Khassi, Ghodratollah Roshanaei

**Affiliations:** ^1^Department of Biostatistics, School of Public Health, Hamadan University of Medical Sciences, Hamadan, Iran; ^2^Research Center for Health Sciences, School of Public Health, Hamadan University of Medical Sciences, Hamadan, Iran; ^3^Department of Epidemiology, School of Public Health, Hamadan University of Medical Sciences, Hamadan, Iran; ^4^Modeling of Noncommunicable Diseases Research Center, Hamadan University of Medical Sciences, Hamadan, Iran; ^5^Behavioral Disease Research Center, Kermanshah University of Medical Sciences, Kermanshah, Iran; ^6^Department of Health, School of Public Health, Kermanshah University of Medical Sciences, Kermanshah, Iran

**Keywords:** HIV, Tuberculosis, Competing risks, Longitudinal outcomes, Joint model

## Abstract

**Background:** Tuberculosis (TB) and human immunodeficiency virus (HIV) are major public health challenges globally, and the number of TB infections and death caused by HIV are high because of HIV/ TB co-infection. On the other hand, CD4 count plays a significant role in TB/HIV co-infections. We used a joint model of longitudinal outcomes and competing risks to identify the potential risk factors and the effect of CD4 cells on TB infection and death caused by HIV in HIV-infected patients.

**Study Design:** This was a retrospective cohort study.

**Methods:** The current study was performed on 1436 HIV+patients referred to Behavioral Diseases Counseling Centers in Kermanshah Province during 1998-2019. In this study, joint modeling was used to identify the effect of potential risk factors and CD4 cells on TB and death caused by HIV.

**Results:** The results demonstrated that the decreasing CD4 cell count was significantly associated with an increased risk of death, while it had no significant relation with the risk of TB. In addition, patients with TB were at a higher risk of death. Based on the results, a significant relationship was found between CD4 count and sex, marital status, education level, antiretroviral therapy (ART), time, and the interaction between time and ART. Further, people infected with HIV through sexual relationships were at higher risk of TB, while those with a history of imprisonment who received ART or were infected with HIV through drug injection had a lower risk of TB.

**Conclusion:** The findings revealed that the decreasing CD4 count had a significant association with an increased risk of death caused by HIV. However, it was not significantly related to the risk of TB. Finally, patients with TB were at higher risk of death caused by HIV.

## Background

 Tuberculosis (TB) and human immunodeficiency virus (HIV) are significant public health challenges worldwide, and the number of deaths associated with both TB and HIV, HIV/TB, and co-infection is abundant.^1^

 In 2019, 39 million people living with HIV were aware of their illness, while 7.1 million people did not know they had HIV. Furthermore, 1.7 million people have recently been infected with HIV, and about 690 000 have died from HIV-related diseases. Iran had the highest HIV prevalence among people who injected drugs in the Middle East in 2010 and 2014 (15.4 and 13.8%, respectively).^2-4^ TB is one of the most common causes of death from infectious diseases. In 2021, approximately 10.6 million people were diagnosed with TB worldwide (including 187 000 people with HIV). The increase in the incidence of TB (new cases per 100 000 population per year) in 2020-2021 was 3.6%, which has decreased by about 2% per year compared to the last decade. Globally, the estimated number of TB deaths increased in 2019-2021. In addition, 1.6 million people, including 187 000 people living with HIV, died of TB in 2021. The number of people newly diagnosed with TB has decreased from 7.1 million in 2019 to 5.8 million in 2020. Further, 703 000 people living with HIV developed TB in 2021, only 46% of whom had access to life-saving antiretroviral therapy (ART).^5-7^

 Studies have shown that TB patients have a low CD4 cell count, and the number of people with a meager CD4 cell count, even less than 200 cells, is high among HIV/TB patients. Therefore, the CD4 count is affected by TB and HIV infections.^8-13^ Although there is no definitive treatment for HIV, ART reduces the progression of the virus in people with HIV.^6^ Studies indicated that the initiation of ART increases the survival of people living with HIV even if they have low CD4 cell counts.^14^ ART also protects HIV patients against TB^15^. Although this therapy reduces the risk of TB, it is not certain that it is also effective in controlling HIV-related TB.^16,17^ As mentioned earlier, HIV/TB is deadly, and the burden of HIV/TB mortality is high in low-income countries. Thus, research is particularly important in reducing the burden of TB in people living with HIV and vice versa. It is also vital to end mortality among people living with HIV.^18,19^

 Considering that HIV-positive people may experience TB or death during the follow-up, the occurrence of TB can no longer be investigated if the death occurs before TB. Furthermore, given that our goal is to examine factors affecting the time to TB (the interest event) in HIV patients, to avoid biasing the results, a competing risk model was used to explore factors affecting the time to TB. It should be noted that CD4 cell counts play a crucial role in both TB and the death of HIV infections. Based on our knowledge, no studies have so far evaluated the effect of CD4 counts on TB infection and death caused by HIV in HIV-infected individuals.

 Hence, in the present study, a joint model of longitudinal outcomes and competing risks was used to identify the potential risk factors and the effect of CD4 cells on TB infection and death caused by HIV in HIV-infected patients.

## Methods

###  Ethics Approval and Consent to Participate

 Written informed consent was obtained from all the participants, and for illiterates and participants under the age of 16, a contest was obtained from parents/legally authorized representatives with confidentiality regarding patients’ names and surnames. This study was approved by the Research Ethics Committee of Hamadan University (No. IR.UMSHA.REC.1398.1066). The study adhered to relevant guidelines and regulations.

###  Data

 Overall, 1436 HIV-infected patients were assessed in this study. Data were collected in the Health Department of Kermanshah, in the west of Iran, from 1998 to 2019. The study was approved by the Research Council of Hamadan University of Medical Sciences. The original dataset consisted of 2034 patients, 598 of whom were excluded because their CD4 cell counts were measured only one time. The analysis was performed on the remaining 1436 patients. One person was considered as an HIV-positive case according to the requirements and definitions of the country and regardless of the clinical stage confirmed by laboratory criteria. In the Islamic Republic of Iran, a person is recognized as an HIV-positive patient if they have a history of two positive enzyme-linked immunosorbent assay (ELISA) tests, one positive Western blot test until a few years ago or a rapid HIV test, and two positive ELISA tests (that one the fourth generation of HIV ELISA test) in recent years. Additionally, a person who has three positive sputum smears or culture tests is known as a person with TB. The World Health Organization defines an immunodeficiency syndrome (AIDS) case as a probable or definitive diagnosis of stage 4 and/or a CD4 cell count of fewer than 200 cells/mm^3^.^20^ Our response variables in this study were the time (in months) from HIV diagnosis to TB (the event of interest) and time to death due to HIV (the competing event). The subject who does not experience these events is known as a censor. The collected information was as follows:

- Demographic information (age at diagnosis, gender, level of education, and marital status);
- Behavioral information (being in prison, drug abuse, and ways of HIV transmission);
- Receiving ART.

Since the diagnosis of HIV infection, the CD4 cell count has been measured during the time. The frequency of CD4 measurements would depend on the survival time. The CD4 cell count of all patients who enrolled in the study was measured at least two times.^20^ The normality of CD4 cells was checked before fitting the model. The results demonstrated that the data on CD4 cell count was completely skewed; thus the fourth root transformation of the CD4 cell count was considered to normalize the data.

###  Joint Model

 A joint model of longitudinal and competing risks comprised of two sub-models, namely, a linear mixed-effects sub-model for longitudinal CD4 cell count and a cause-specific hazard sub-model for the competing risks of TB infection and death caused by HIV. It should be noted that random effect variables link the two sub-models.

###  Longitudinal Sub-model

 It is assumed that *Y*_ij_ is the longitudinal outcome measured at time j for subject i, *i* = 1,2,…,*m* and, *j* = 1,2,…,*n*_i_, where *n*_i_ is the number of observations for individual i. The linear mixed-effects model is defined as follows:


(1) $$Y_{ij}=X_{ij}^{T}\beta +Z_{ij}^{T}b_{i}+\varepsilon_{ij}$$

where **X**_ij_, β, and **Z**_ij_ represent a *p*×1 vector of fixed effects, the vector of coefficient of fixed effects, and a *q*×1 vector of random effects $b_{i},\;b_{i}∼N_{q}\left(0,\sum_{b}\right)$ , respectively. Moreover, $\varepsilon_{ij}∼N\left(0,\sigma^{2}\right)$ is the measurement error.

###  Competing Risks Sub-model

 A cause-specific hazards sub-model was used to model competing risks. Let *C*_i_*= ( T*_i_*,D*_i_*)* be the competing risks data on subject i, where *T*_i_ is the failure/censoring time and *D*_i_ denotes the type of failure, $D_{i}\in \left\{0,1,...,g\right\}$ . Paying attention to *D*_i_ = 0 indicating a censored event and *D*_i_ = *l* shows that subject i fails from the *l*th type of failure, where * l* = 1,…, g. Two events were considered in the present study. The model is provided as follows:


(2) $$\lambda_{l}\left(t;X_{i}\left(t\right),u_{i},\gamma_{l},\nu_{l}\right)=\lambda_{0l}\left(t\right)\exp\left\{X_{i}{\left(t\right)}^{T}\gamma_{l}+\nu_{l}u_{i}\right\}$$

The function $\lambda_{l}\left(t;X_{i}\left(t\right),u_{i},\gamma_{l},\nu_{l}\right)$ is an instantaneous hazard function for the failure of type *l* at time t given the vector of covariates *X*_i_*(t)* and the frailty *u*_i_ in the presence of all other failure types. *λ*_0l_*(t)* represents a baseline hazard function for risk *l*, where *l* = 1,…, g.*v*_l_ The vector is the correlation estimate between different risks. It is assumed that *b*_i_ and *u*_i_ jointly have a multivariate normal distribution:


(3) $$\theta_{i}=\left(\begin{array}{l} b_{i}\\ u_{i} \end{array}\right)∼N\left(\left(\begin{array}{l} o\\ o \end{array}\right),\left(\begin{array}{l} \sum_{bb}\sum {}_{bu}^{T}\\ \sum_{bu}\sigma_{u}^{2} \end{array}\right)\right)$$

An expectation-maximization (EM) algorithm was used to estimate the parameters.^21^ All analyzes were performed at a significant level of 0.05 using the JMcmprsk library in R software, version 3.6.1.

## Results

 In this study, 1436 HIV patients were followed up, of which 85.6% were infected with TB and 11.1% of them experienced death due to HIV infection. The mean follow-up time for TB and death due to HIV was 93.09 and 229.22 months, respectively. The frequency of CD4 cell counts varied between 2 and 17 times during the follow-up period, but the number of the measured CD4 counts was considered between 2 and 12 because the CD4 cell count was measured at a maximum of 12 times in most patients. The demographic information of HIV patients is presented in Table 1. The mean (standard deviation) age of patients at diagnosis was 34.23(37.01) years. Most patients were males (80.8%) and married (56.3%) and had a low level of education (96.7%) and a history of imprisonment (70%). Further, 76.3% of patients were drug users, and 60.2% of them received ART. Nearly 65% of people became infected with HIV through drug injection.

**Table 1:** The characteristics of HIV-infected patients

**Variables**	**Frequency**	**Percent**
Gender		
Male	1161	80.8
Female	275	19.2
Marital status		
Single	627	43.7
Married	809	56.3
Educational level		
High (academic)	47	3.3
Low (illiterate and school)	1389	96.7
Imprisonment		
No	431	30.0
Yes	1005	70.0
Drug abuse		
No	341	23.7
Yes	1095	76.3
Antiretroviral therapy		
No	572	39.8
Yes	864	60.2
Sexually transmitted HIV infection		
No	1127	78.5
Yes	309	21.5
Drug injection transmitted HIV		
No	498	34.7
Yes	938	65.3
Total number	1436	

*Note*. HIV: Human immunodeficiency virus.

 The joint model was fitted to investigate the effect of different covariates on cause-specific hazards, TB infection and death caused by HIV, and the trend of CD4 cell count, the results of which are provided in Table 2.

**Table 2:** The results of the joint model for competing risks (TB/death caused by HIV) and longitudinal measurements (CD4 cell count)

**(A) Longitudinal Endpoint**	**Parameter**	**SE**	***P*** ** value**
Age (y)	-0.00	0.00	0.761
Gender (Male/female)	-0.12	0.03	0.001
Marital status (Married/single)	-0.09	0.03	0.003
Education (High/low)	0.17	0.08	0.026
Antiretroviral therapy (Yes/No)	0.84	0.04	0.001
Time (mon)	0.09	0.00	0.001
Antiretroviral therapy × time^a^	0.02	0.00	0.001
(B) Survival endpoint			
**Risk 1: TB infection**	**Parameter (SE)**	** HR (95% CI)**	***P*** ** value**
Age (Year)	0.00 (0.00)	1.00 (0.10,1.01)	0.849
Gender (Male/female)	0.21 (0.18)	1.23 (0.87,1.75)	0.240
Marital status (Married/single)	0.05 (0.10)	1.05 (0.87, 1.27)	0.592
Education (High/low)	0.14 (0.31)	1.15 (0.63,2.09)	0.653
Sexually transmitted HIV infection (Yes/No)	0.33 (0.15)	1.39 (1.03, 1.88)	0.033
Drug injection transmitted HIV (Yes/No)	-0.49 (0.13)	0.62 (0.48,0.79)	0.001
Drug abuse (Yes/No)	0.18 (0.19)	1.20 (0.83, 1.72)	0.329
Antiretroviral therapy (Yes/No)	-0.55 (0.10)	0.58 (0.47,0.70)	0.001
Imprisonment (Yes/No)	-0.63 (0.15)	0.53 (0.40,0.71)	0.001
**Risk 2: Death caused by HIV**	**Parameter (SE)**	** HR (95% CI)**	***P v*** **alue**
Age (y)	-0.00 (0.01)	0.10 (0.98, 1.02)	0.922
Gender (Male/female)	-1.00 (0.42)	0.37 (0.16,0.84)	0.017
Marital status (Married/single)	-0.17 (0.18)	0.84 (0.59,1.21)	0.344
Education (High/low)	0.19 (0.69)	1.21 (0.31, 4.70)	0.781
Sexually transmitted HIV infection (Yes/No)	-0.68 (0.54)	0.51 (0.17,1.46)	0.208
Transmission of HIV by injection (Yes/No)	0.19 (0.38)	1.21 (0.57,2.56)	0.614
Imprisonment (Yes/No)	-0.48 (0.33)	0.62 (0.33, 1.17)	0.143
Drug abuse (Yes/No)	0.60 (0.59)	1.82 (0.57, 5.84)	0.312
Antiretroviral therapy (Yes/No)	-2.58 (0.25)	0.08 (0.05,0.12)	0.001
**(C) Random effects**	**Parameter (SE)**		***P *** **value**
$\sigma_{b_{1}u}$	0.16 (0.39)		0.682
$\sigma_{b_{2}u}$	-6.78 (0.90)		0.001
$\nu_{2}$	0.32 (0.15)		0.032

*Note*. HIV, Human immunodeficiency virus; HR, Hazard ratio; SE, Standard Error; CI, Confidence interval. ^a^ Interaction term.

 Based on the results of evaluating the effect of covariates in the longitudinal model of CD4 cell count (Table 2A), the time effect estimate was positive, indicating that the average fourth root of CD4 cells increases with increasing time (β = 0.09, *P* < 0.001). Estimating the effect of sex was negative, implying that the mean fourth root of the CD4 cell count in men was 0.12 units lower than in women (β = -0.12, *P* < 0.001). Marital status also had a significant negative effect on the mean fourth root number of CD4 cells (β = -0.09, *P* = 0.003). The interaction between time and ART was positive and significant, which showed that the effect of ART on the CD4 cell count changes over time (β = 0.02, *P* < 0.001). Moreover, antiretroviral treatment had a significant positive effect on the mean of the fourth root of the CD4 cell count so that people who received ART had a higher mean of the fourth root of the CD4 cell count than those who received no treatment (β = 0.84, *P* < 0.001). The results revealed that education could also significantly affect the mean of the fourth root of the CD4 cell count (β = 0.17, *P* = 0.026).

 The results obtained from fitting the competing risks model are presented in Table 2B. As shown, HIV transmission methods, prison history, and ART had a significant impact on the risk of TB. According to the results, a significant relationship was found between having a prison history and TB so that the TB hazard ratio (HR) in people with a history of imprisonment was 47% lower than those who had no history of imprisonment (HR = 0.53, *P* < 0.001). ART also had a significant effect on the risk of TB so that the HR was 42% lower in patients who received ART treatment than in those who did not receive any treatment (HR = 0.58, *P* < 0.001). Based on the findings, HIV transmission through drug injection had a significant relationship with TB so that patients with HIV through drug injection were 38% less at TB risk than those infected through other ways (HR = 0.62, *P* < 0.001). People who became infected with HIV through sexual relationships were 39% more likely to develop TB than those who were not (HR = 1.39, *P* = 0.033). The results demonstrated that ART was significantly associated with death caused by HIV so that people who received ART were 92% less at risk of death caused by HIV than those who received no treatment (HR = 0.08, *P* < 0.001). Additionally, men were 63% less at risk of death caused by HIV than women (HR = 0.37, *P* = 0.017).

 Estimating v_2_ = 0.32 in the model’s random effects represented a significant positive relationship between competing risks of TB anddeath caused by HIV (*P* = 0.032), confirming that patients with TB are more in danger of death caused by HIV. Furthermore, the estimate $\sigma_{b_{2}u}$ =-6.78 suggests a significant relationship between the mean of the fourth root of the CD4 cell count and death caused by HIV. This implies that patients with fewer CD4 cells have a higher risk of death caused by HIV. However, no significant relationship was found between the CD4 cell count and TB.

## Discussion

 The present study evaluated the effect of several covariates and CD4 cell counts on TB and death caused by HIV using a joint model of longitudinal outcomes and competing risks.

 The findings indicated that patients with TB are at higher risk of death caused by HIV, which is consistent with the results of other studies.^22,23^

 Likewise, patients with fewer CD4 cells had a higher risk of death caused by HIV. In other words, the risk of death caused by HIV increased by reducing the average fourth root of CD4 cell counts. These results are in line with those of other studies, representing that a low initial CD4 cell count at the time of diagnosis or a decreasing trend over time is associated with an increased risk of death caused by HIV.^24-26^ The findings showed that patients who received ART over time had an increasing trend in the mean fourth root of the CD4 cell count. According to the results, antiretroviral treatment had a significant positive effect on the mean of the fourth root of CD4 cell count so that those receiving ART had a higher mean of the fourth root of the number of CD4 cells than those who did not receive the treatment. The results from a systematic review, including randomized controlled trials and observational studies, revealed that initiating ART in patients with a lower CD4 cell count increased CD4 cell counts and stopped increasing virus load.^27^

 Based on the results, marital status affected the fourth root of the CD4 cell count so that married people had fewer cells than single people, which contradicts the findings of other studies. This could be because the average age of married people in this study was higher than that of single people.^28,29^

 In this study, sex had a significant effect on the fourth root of the CD4 cell count, which corroborate with the results of some other studies.^30,31^

 The results demonstrated that the trend of changes in the mean of the fourth root of the CD4 cell count increased in people who received ART over time. This finding conforms to that of a study by Chitra et al, indicating that ART intake increased CD4 levels in people with low CD4 levels.^32^ Based on the present study results, time had a positive and significant effect on the mean of the fourth root of the CD4 cell count, which is in line with the result of another study.^31^

 Higher education had a positive effect on the fourth root mean of the CD4 cell count. This may be because people with higher education are less likely to become infected with HIV a result consistent with the Gergiso and Erango study.^33^

 The findings showed a significant relationship between TB and imprisonment, and people with a history of imprisonment had a lower risk of TB, which is inconsistent with those of other studies, demonstrating that the risk of TB was higher in prisoners.^34-36^ The obtained data confirmed that ART could significantly reduce the risk of TB so that the risk of reducing TB in patients who received treatment was 42% lower than in patients who received no treatment. This result is in conformity with the results of other studies, although some studies reported contrary results in this regard.^37-40^

 The present study results also indicated that sexual transmission increases the risk of TB, which does not match the results of other studies.^41,42^ Many studies reported a significant association between HIV transmission through drug injection and TB; some of them had a higher risk of developing TB through drug injection and others, in line with the findings of the present study, mentioned a negative relationship between drug injection and TB.^43-46^

 Similarly, Bahakeem found that the risk of death caused by HIV in HIV patients was still high using flexible models and gradually decreased after the onset of ART.^47^ Other studies have shown the effectiveness of ART reception on the survival time of patients with HIV and AIDS,^48,49^ which is consistent with the findings of the present study.

 Based on the results of the current study, men had a lower risk ofdeath caused by HIV among HIV patients than women, which contradicts the findings of Liu et al; they concluded that men were more likely to die than women.^50^

 This study had some limitations. Due to the use of retrospective data collected by data centers, it was impossible to verify the accuracy of data, which could be biased. Another limitation was the number of CD4 cells; some people had a low repetition, while we know that it is better to have a higher number of CD4 cell replications. However, a joint model was developed to investigate the simultaneous effect of CD4 cell counts on the risk of TB and death caused by HIV, which may help reduce mortality in people living with HIV and TB.

**Highlights**

- The decreasing CD4 cell count was significantly associated with an increased risk of death but was not significantly related to the risk of TB.
- Patients with TB were at a higher risk of death.
- There was a significant relationship between CD4 cell count and sex, marital status, education level, ART, time, and the interaction between time and ART.
- People infected with HIV through sexual relationships were also at higher risk of TB.
- Those with a history of imprisonment who received ART or were infected with HIV through drug injection had a lower risk of TB.

## Conclusion

 Using joint modeling, it was possible to simultaneously examine the effect of covariates on CD4 cell counts, the risk of TB, and death caused by HIV. In summary, the results revealed that the decreasing trend of the CD4 cell count was significantly related to an increased risk of death caused by HIV, while it was not significantly associated with the risk of TB. The results also showed that patients with TB infection were at higher risk of death caused by HIV.

## Acknowledgements

 This study was adapted from the PhD thesis at Hamadan University of Medical Sciences. We would like to thank the Vice-Chancellor of Research and Technology, Hamadan University of Medical Sciences for the approval and support of the study.

## Competing Interests

 The authors declare no conflict of interests.

## Funding

 The study was funded by the Vice-chancellor for Research and Technology, Hamadan University of Medical Sciences (Grant No. 9812209740).
